# Occurrence of Furfural and Its Derivatives in Coffee Products in China and Estimation of Dietary Intake

**DOI:** 10.3390/foods12010200

**Published:** 2023-01-02

**Authors:** Qing Liu, Pingping Zhou, Pengjie Luo, Pinggu Wu

**Affiliations:** 1 Key Laboratory of Food Safety Risk Assessment, Ministry of Health, China National Center for Food Safety Risk Assessment, Beijing 100021, China; 2 Zhe Jiang Provincial Center for Disease Control and Prevention, Hangzhou 310051, China

**Keywords:** furfural, coffee, exposure, GC-MS technology

## Abstract

This is the first report on the content of furfural and its derivatives in coffee products in China. The concentrations of furfural and its derivatives in 449 sampled, commercially available coffee products in China were analyzed through a GC-MS technique, and the associated health risks were estimated. As a result, 5-hydroxymethyl furfural (5-HMF) was identified as the predominant derivative compound, with the highest concentration of 6035.0 mg/kg and detection frequency of 98.7%. The mean dietary exposures of 5-HMF, 5-MF(5-methylfurfural), and 2-F(2-furfural) in coffee products among Chinese consumers were 55.65, 3.00, and 3.23 μg/kg bw/day, respectively. The ranges of mean dietary intake of furfural and its derivatives based on age groups were all lower than the acceptable daily intake (ADI) and the toxicological concern threshold (TTC). Risk evaluation results indicate that coffee product intake did not pose potential risks to consumers. Notably, the analysis revealed that children aged 3–6 years had the highest mean exposure due to their low body weight.

## 1. Introduction

Furfural and its derivatives are considered as contaminants, naturally occurring compounds, and vital food constituents. They are found in various foods, such as coffee, fruit, honey, vinegar, breakfast cereals, baked bread, and milk [1,2,3,4,5]. Furfural and its derivatives are found in mg/kg quantities in various foods [6]. Their generation is mainly via caramelization and Maillard reactions, which produces the desirable colors and flavors of heat-processed foods. However, over-processing can also lead to the loss of nutritional value and the damage of vital food constituents, generating harmful substances, such as furfural, furan, and acrylamide [2,3,7,8]. 

Despite numerous available studies of furfural and its derivatives, 5-HMF is the most reported compound. The generation of 5-HMF can be used as a food quality indicator [9]. It could also be a signal of the relevant contamination risk during heat processing. According to the general knowledge of Maillard chemistry, particularly the caramelization reaction, fructose and glucose are the main sources of 5-HMF formation [10,11]. The accumulation of 5-HMF is crucial for acrylamide formation in coffee production [12].

HMF has an oral LD_50_ (lethal dose) of 3.1 g/kg body weight in rats [13,14]. Cytotoxic, genotoxic, mutagenic, and carcinogenic properties of 5-HMF in rats and mice have been reported previously [15,16,17,18]. An experimental study revealed that 5-HMF can also produce certain genotoxicity and cytotoxicity to the human body and reduce the activity of related enzymes [19]. However, data from epidemiological studies or case reports on the potential association of HMF with cancer risk in humans are not available. As a result, the International Agency for Research on Cancer (IARC) has classified furfural as Group 3 due to limited evidence of its carcinogenicity for humans.

Coffee has been regarded as the main source of 5-HMF [20]. Coffee consumption is a popular dietary habit in Western cultures, which enhances the exposure to furfural and its derivatives [21,22]. Numerous countries have developed detection methods for furfural and its derivatives in coffee. However, these methods are mainly limited to the detection of 5-HMF, rather than the simultaneous analysis of 5-MF and 2-F. The quantification of 5-HMF levels in coffee products has been extensively reported. Although coffee products are becoming increasingly popular, especially among the young population in China, the consumption of furfural and its derivatives through coffee products has not been studied. The levels of furfural and its derivatives only were previously determined for infant formulas, vinegar, wine, and milk from China [4,23,24,25,26,27]. Reliable data on the incidence and contamination levels of furfural and its derivatives in coffee products are required to enable exposure and risk assessments.

Therefore, this work aimed to develop a convenient, accurate, rapid, and easy GC-MS technology for analyzing furfural and its derivatives in coffee products collected from China. Consumers’ daily intake and related health risks via food consumption were then calculated and assessed. According to our knowledge, this is the first report to study the occurrence of furfural and its derivatives in coffee products.

## 2. Materials and Methods

### 2.1. Chemicals and Reagents

The 5-hydroxymethyl furfural (>99%), 5-methylfurfural (>99%), and 2-furfural (>99%) were purchased from Chem Inc. (Augsburg, Germany). D_4_-2-furfural (>99%) and ^13^C_6_-5-hydroxymethyl furfural (>99%) internal standards were purchased from Toronto Research Chemicals Inc. (TRC, Toronto, Canada). Acetonitrile (>99%), sodium carbonate, and ethyl alcohol were obtained from Sinopharm (Beijing, China; AR).

### 2.2. Standard Solution Preparation

The standard 0.5 mg/mL stock solutions of 5-HMF, 5-MF, and 2-F were prepared by dissolving 25 mg of each compound with acetonitrile in a 50 mL flask, while the standard solutions were obtained based on serially diluting the corresponding intermediate solutions with acetonitrile at 25 or 5.0 μg/mL concentrations. Two stocks of internal standard solutions (D_4_-2-F, 2.5 mg/mL and ^13^C_6_-5-HMF, 0.1 mg/mL) were prepared by dissolving 25 mg of D_4_-2-F and 1 mg of ^13^C_6_-5-HMF in acetonitrile into a 10-mL volumetric flask, respectively. Mixed standard solutions at 10 μg/mL were obtained by mixing 40 μL of 2.5 mg/mL D_4_-2-F or 1 mL 0.1 mg/mL ^13^C_6_-5-HMF with acetonitrile in a flask, which was stored at −20 °C in darkness.

### 2.3. Sample Collection and Preparation

Up to 449 samples of coffee products from the most well-known brands were collected from the major markets in China in 2021, which included 67 coffee beans, 42 coffee powders, 34 instant coffee powders, 122 drink powders, 72 packaged coffee drinks, and 112 takeaway coffees. Samples were collected from 14 major Chinese regions, including Hebei, Inner Mongolia, Liaoning, Fujian, Hubei, Sichuan, Beijing, and Henan. The coffee products included major domestic and imported brands from Italy, Japan, Malaysia, and Indonesia. The takeaway coffees were collected and stored at 4 °C. Other samples were stored at room temperature and analyzed within one month. The roasted coffee beans were ground using a food grinder (Ika Tube Mill, Aachen, Germany city, state, country; CS025) to powder and then sifted through a 60-mesh filter and stored at −4 °C.

### 2.4. Sample Preparation

#### 2.4.1. Sample Extraction

Coffee beans or powder products: Approximately 0.5 g roasted coffee beans or coffee powder products, 5 mL 50% ethanol, and 5 mL 10% Na_2_CO_3_ were added to a 50 mL polypropylene tube. The tube was vortexed for several seconds before being ultrasonicated in a water bath for 10 min. After this, we transferred the extraction solution to a 25 mL volumetric flask with ultrapure water. After discarding the first five drops, the mixture was shaken and filtered with filter paper into a 50 mL polypropylene tube.

Instant coffee powder: Approximately 0.3 g instant coffee powder, 5 mL 50% ethanol, and 5 mL 10% Na_2_CO_3_ were added to a 15 mL polypropylene tube. The tube was vortexed for several seconds, and the extraction solution was transferred into a 100 mL volumetric flask containing ultrapure water. The mixture was shaken and filtered with filter paper into a 50 mL polypropylene tube after discarding the first five drops.

Drinking powder (using food additives): Approximately 0.5 g drink powder, 2 mL 50% ethanol, and 5 mL 10% Na_2_CO_3_ were spiked in a 10 mL polypropylene tube. The tube was vortexed for several seconds. The extraction solution was transferred into a 10 mL volumetric flask containing ultrapure water.

Packaged coffee drinks and takeaway coffee: Approximately 2.5 mL packaged coffee drink or takeaway coffee and 5 mL 10% Na_2_CO_3_ were spiked in a 15 mL polypropylene tube. The tube was vortexed for several seconds. The extraction solution was transferred into a 10 mL volumetric flask containing ultrapure water.

#### 2.4.2. Sample Purification

Firstly, 1.5 mL aqueous supernatant was transferred to a 15 mL polytetrafluoroethylene (PTFE) centrifuge tube, and 0.15 mL of 10 μg/mL furfural internal standard and 1.5 mL of acetonitrile were then added. Subsequently, 0.6 g sodium chloride (NaCl) was added and vortexed for 5 min. The sample was then centrifuged for 2 min at 10,000 rpm based on a desktop centrifuge (RJ-GL-1850; Anting Factory, Shanghai). The 1 mL supernatant layer was cleaned with a QuEChERS tube with 300 mg/2 mL PG-004 (containing C18 adsorbent, SCX adsorbent, and Na_2_SO_4_), vortexed for 1 min, and centrifuged for 2 min at high speed. Then, the supernatant was taken and filtered using a 0.22 μm PTFE filter membrane before GC-MS/MS testing.

### 2.5. GC-MS Analysis

The GC-MS testing was performed on an Agilent 8890 GC system connected to an HES EI source (Agilent, Santa Clara, CA, USA). An HP-Innowax column was applied for separation and method validation.

The program temperature was set as follows: initial temperature held at 60 °C for 2 min, keep heating to 130 °C at speed of 10 °C/min, and then heat to 240 °C at 30 °C/min, held for 5 min, and finally held at 250 °C for 10 min. 

The mass detector was operated at 70 eV in single-ion monitoring (SIM) mode. The temperatures of the injection, transfer line, and ion source were 220, 240, and 230 °C, respectively. The injection mode was splitless with an injection volume of 1μL, and the carrier gas was helium (purity >99.999%), flowed at a constant rate of 1 mL/min. The solvent delay was 6 min. SIM of target ions was performed according to the parameters listed in Table 1.

### 2.6. Quantification of Furfural and Its Derivatives

We took 10, 20, 50, and 200 μL of 5 μg/mL standard mixed solution and 100, 400, and 800 μL of 25 μg/mL mixed standard solution, added 100 μL of 10 μg/mL mixed internal standard solution, respectively, and finally added acetonitrile to 1 mL to obtain 0.05, 0.1, 0.25, 1.0, 2.5, 10.0, and 20.0 μg/mL mixed standard solutions, including 1.0 μg/mL internal standard. Calibration curves were obtained based on plotting the peak area and concentrations via an internal standard method by GC-MS.

### 2.7. Method Validation

Certified quality control laboratories for chemical detection by China National Center for Food Safety Risk Assessment (CFSA) were applied to ensure the monitoring data’s accuracy. Isotope-labeled internal standards were applied for quantification.

Coffee products are rich in nutrients, which include a large amount of furfural and its derivatives. In this case, if there is no appropriate matrix blank (for all or some analyses) in the research process for analysis, the low-concentration standard addition analysis could be carried out. Taking this fact into account, we selected low-background coffee product samples for comparative analysis. In the process of recovery rate research, the standard addition method based on furfural concentration was selected for the analysis by integrating various factors, and the results were statistically processed to provide support for subsequent research. The relative standard deviation (RSD) was calculated by spiking the low-background furfural matrices with standard solutions at 0.1, 0.5, and 1.5 μg/mL for coffee samples. Prepared samples were analyzed on the same day. The recoveries were set to 70–110%, and the RSDs were between 7.4 and 8.9%.

The system linearity was assessed based on seven concentration points within −0.05–20 μg/mL for furfural and its derivatives. R^2^ was greater than 0.998 for each analysis. The limit of detection (LOD) and limit of quantitation (LOQ) were determined with S/N ratios at 3 and 10. The LODs and LOQs of 5-HMF in liquid coffee products (packaged coffee drinks and takeaway coffee) were 0.1 mg/L and 0.3 mg/L. The LODs and LOQs of 5-HMF, 5-MF, and 2-F in solid coffee (coffee beans, coffee powder, and drinking powder) were 2.0 and 5.0 mg/kg, respectively. Levels < LOD were deemed as 1/2 LOD during mean analysis.

### 2.8. Coffee Consumption Data

The data related to coffee consumption in China from 2013 to 2014 were obtained based on the results of a survey of China’s national alcoholic beverages at this time via multi-stage random cluster sampling. A total of 32 sampling sites were set up in 16 provinces in China, and the corresponding data were preprocessed to remove invalid or obviously abnormal data, so as to improve the application value of the results. A face-to-face 24-h diet interview was conducted on one weekend day (Saturday or Sunday) and two weekdays. The interval between two interviews was no less than 5 days, with a total of three interviews. In this survey, we included 309 subjects, whose ages ranged from 3 to 80 years old, and all had a certain history of coffee consumption. The coffee products selected during the study mainly included coffee beans, coffee powder, and coffee drinks. After sorting out the collected data, the results were as shown in Table 2. The average daily consumption was 7.8, 4.8, and 74.2 g/day for coffee beans, coffee powder, and liquid coffee drinks, respectively, for the Chinese adult population. 

### 2.9. Calculation of the Estimated Daily Intake

The dietary exposure was calculated by summing the coffee consumption information with the mean furfural and its derivatives’ concentrations. The summed value was regulated according to each consumer’s body weight (BW) [28]. Subsequently, the relevant total dietary exposure to furfural and its derivatives was estimated for each consumer. The exposure can be obtained based on the formula

(1) $$Exp_{i}=\overset{p}{\underset{k=1}{\sum }}\frac{Com_{ik}\times Con_{k}}{BW_{i}}$$

in which *Exp_i_* refers to the dietary exposure to furfural and its derivatives of consumer *i* for coffee (μg/kg bw/day); *Com_ik_* represents the consumption of consumer *i* of coffee product *k* (g/d); *Con_k_* represents the mean concentration of furfural and its derivatives (mg/kg); *BWi* represents the body weight of consumer *i* (kg); pi refers to the number of coffee products of consumer *i*.

This study estimated the exposure to furfural among the Chinese population. The population was divided into three age categories, including 3–6, 7–17, and ≥18 years old.

Based on relevant, widely accepted evaluation methods, furfural and its derivatives’ exposure levels were compared with the established ADI level of 0.0–0.5 mg/kg bw/d by EFSA [29]. In addition, the European Food Safety Authority Study Group on Animal Feed Additives and Products (FEEDAP) classifies 5-MF as a Cramer Class II chemical with a concern threshold of 9 μg/kg bw based on the TTC for evaluating safety in animal feeds. Generally, dietary exposure lower than those of ADI or TTC indicated a low health risk, while higher exposure values required further attention.

### 2.10. Statistical Analysis

All data analysis was carried out via R-4.0.5 software(R Foundation for Statistical Computing, Vienna, Austria; http://www.R-project.org/ (accessed on 26 October 2022)). For derivatives and outliers, different sample groups were analyzed using Spearman’s correlation and a non-parametric Kruskal–Wallis test based on a significance threshold of 0.05.

## 3. Results and Discussion

### 3.1. Concentrations of Furfural and Its Derivatives in Coffee Products

**Figure 1:**
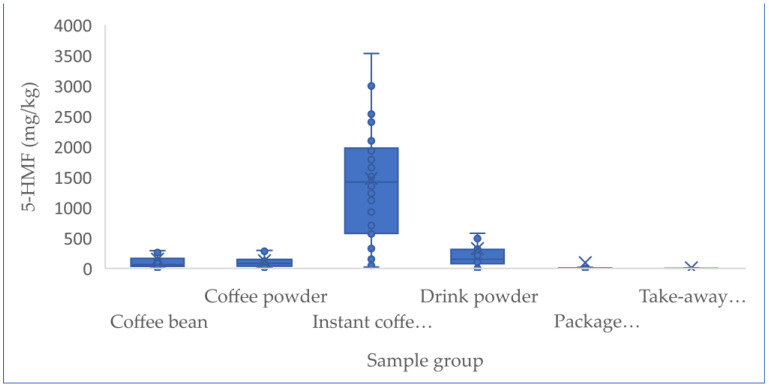
Box plots comparing 5-HMF concentrations in 6 coffee products in 2021. Lower and upper ends of the box represent the lower and upper quartiles (25th and 75th percentiles), the middle line represents the median, and the whiskers represent the minimum and maximum, excluding outliers.

**Figure 2:**
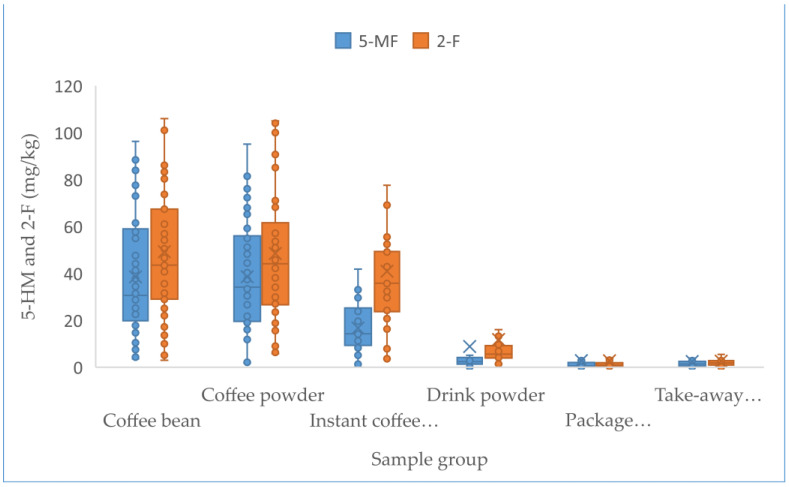
Box plots comparing 5-MF and 2-F concentrations in 6 coffee products in 2021. Lower and upper ends of the box represent the lower and upper quartiles (25th and 75th percentiles), the middle line represents the median, and the whiskers represent the minimum and maximum, excluding outliers.

**Figure 3:**
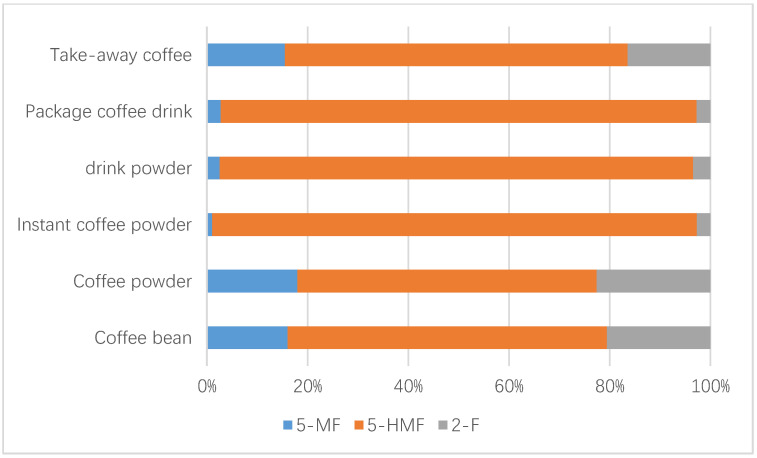
Contributions (%) of 5-MF, 5-HMF, and 2-F to the total furfural and its derivatives’ concentrations for each coffee product.

Up to 449 samples from the Chinese market were studied, including 67 coffee beans, 42 coffee powder products, 34 instant coffee powders, 122 drink powders, 72 packaged coffee drinks, and 112 take-out coffee products. Table 3 describes the sample results of the above analyzed factors, such as the coffee sample number and the concentrations of furfural and its derivatives. Statistical analysis revealed that the concentrations of furfural and its derivatives in coffee beans (7.3–897.0 mg/kg for 5-HMF, 3.5–96.3 mg/kg for 5-MF, 3.0–219.0 mg/kg for 2-F), coffee powder (9.1–651.0 mg/kg for 5-HMF, 1.9–95.1 mg/kg for 5-MF, 5.8–122 mg/kg for 2-F), and instant coffee powder (23.7–5062 mg/kg for 5-HMF, ND-418 mg/kg for 5-MF, 3.3–172 mg/kg for 2-F) were significantly higher than others (adjusted *p* < 0.005), followed by the drink powder (ND-3675.0 mg/kg for 5-HMF, ND-151.0 mg/kg for 5-MF, 1.3–99.0 mg/kg for 2-F). No obvious difference (adjusted *p* > 0.05) existed between the levels of furfural and its derivatives in packaged coffee drinks and takeaway coffee, which both had nearly 4–20-fold lower content relative to those observed in the above coffee samples (adjusted *p* < 0.005), likely due to the added water during processing. The concentrations of 5-HMF in instant coffee powder products ranged from 23.7 to 5062 mg/kg, and were obviously higher (adjusted *p* < 0.005) compared to those in coffee beans and ground coffee powder products. In contrast, the concentrations of 5-MF and 2-F in three coffee products were not obviously different (adjusted *p* > 0.05). No obvious statistical differences in furfural and its derivatives existed among coffee beans and ground coffee powder products, which might be due to the fact that they only involve the physical shredding of coffee beans into coffee powder and thus result in no change in the furfural content. Figure 1 and Figure 2 show the concentrations of furfural and its derivatives in a box plot. The proportions of 5-MF, 5-HMF, and 2-F in relation to the sum of furfural and its derivatives were calculated based on the mean values of all the 449 tested coffee products (Figure 3). Here, 5-HMF was found as the predominant component in all six types of coffee products. In general, the levels of furfural and its derivatives found in this study are substantially lower than those reported in other studies, in which the reported concentrations in roasted coffee ranged from 300 to 2900 mg/kg [30]. For a total of 20 medium roasted coffees in Turkey, the 5-HMF levels ranged from 168.9 to 353.5 mg/kg, with an average value of 260.5 mg/kg [31]. The range of 5-HMF in 108 coffee samples was 51-1143 mg/kg in Korea [22].

Similar levels to those observed in our study were found in commercially roasted coffee beans, which contained 77.7–322, 157–209, and 109–200 mg/kg of 5-HMF, 5-MF, and 2-F, respectively [32]. An analysis of Arabica coffee revealed a 5-HMF concentration range of 51.21–829.52 mg/kg. The study also detected levels of 5-HMF in the range of 24–4023 mg/kg in coffee products [21]. These variations could be due to the differences in the sources of ground coffee, post-harvest processing, extraction methods, and roasting processes [22,33,34].

A previous study found significantly higher 5-HMF content in coffee substitutes and instant coffee [35], which is in accordance with this research. Our results showed that 5-HMF increased with the enhanced degree of concentration of the instant coffee spray (versus roasted beans and powders), while 5-MF and 2-F remained unchanged, which is similar to observations made by Arribas et al. (2010) [21] and Villalón-López et al. (2018) [36]. Roasting conditions and extraction methods are significant factors affecting the volatile compounds in coffee [37]. In the process of producing instant coffee powder from coffee beans and coffee powder, a large amount of 5-HMF is formed [22,38,39].

### 3.2. Estimation of Daily Intake and Risk Assessment

The dietary exposure to furfural and its derivatives from coffee products is listed in Table 4. The average dietary exposure of 5-HMF, 5-MF, and 2-F from coffee products among consumers was 55.65, 3.00, and 3.23 μg/kg bw/day. In the high exposure (P95) group, exposure to 5-HMF, 5-MF, and 2-F was estimated to be 162.96, 8.78, and 9.93 μg/kg bw/day among coffee consumers. The mean dietary exposure levels in various age groups were 1.12–272.67, 0.07–16.15, and 0.07–16.15 μg/kg bw/day, whereas their content in the P95 groups was in the range of 108.91–173.86, 5.70–9.63, and 6.08–10.39 μg/kg bw/day, respectively.

Liquid coffee drinks were the main contributors of 5-HMF, 5-MF, and 2-F exposure for the Chinese consumer, followed by coffee powder (Table 5). Liquid coffee drinks were the major contributor of furfural and its derivatives due to their greater consumption in terms of volume and frequency. In addition, exposure to furfural and its derivatives among adolescents in this study was mainly from liquid coffee, which was lower than that observed in other countries [20,21,40]. Arribas-Lorenzo et al. (2010) [21] only considered the scenario for adult consumption and not children and adolescents. Moreover, some Chinese parents may buy packaged coffee drinks for their children, because some packaged coffee drinks taste better due to the addition of milk and sugar [41]. Furthermore, children have a lower weight, so, compared with adults, children experience higher furfural exposure per kilogram of body weight. Although the levels of furfural in coffee beans were high, the estimated daily intake was negligible for a low level of coffee beans. Numerous studies have focused on 5-HMF contamination because its levels are incidentally higher than those of 5-MF and 2-F [3,39,42].

The observed dietary exposure to furfural and its derivatives in this research was lower than in other countries. For example, coffee was the main source of 5-HMF in the diet, with intake of 70 μg/kg bw/d [20] and 75.15 μg/kg bw/d [21], in the Spanish market, which are approximately 30% higher than the observed values in this study. Other research indicated that coffee accounted for approximately 50% of 5-HMF exposure in Spain [21] and approximately 63% in Norway [40].

Several studies have shown that coffee is the leading source of 5-HMF. As an estimate, high exposure could reach 400 mg/kg bw/din Germany [2]; however, coffee was not the major exposure source of 5-HMF in the assessment study in Germany. According to the latest worldwide consumption data, coffee consumption in China is lower compared to that in European countries, the USA, and Canada. It is likely that varying dietary behaviors determine the differences in exposure to furfural and its derivatives from coffee in different countries.

As shown in Table 4, 2-F exposure levels from coffee products were below the set acceptable daily intake (ADI) in China. The maximum intake level was 16.15 μg/kg bw/day, which equated to only 3.2% of the ADI by EFSA. The average (3.00 μg/kg) and 95th (8.78 μg/kg) percentile intake levels of 5-MF were also lower than the FEEDAP limit of 9 μg/kg bw/day.

A previous study detected a tolerable daily intake (TDI) of 132 mg/day for 5-HMF based on a 40-fold safety margin [43]. This study detected an average daily intake of 3.3 mg/day (55.65 μg/kg bw/day), which is 40-fold lower compared to the TDI value above. The mean exposure of 3.3 mg/day estimated in this study is also much lower than that reported by the FDA, which changes from 4 to 30 mg 5-HMF/person/day [44]. Under this level, no toxic effects occurred at daily doses between 80 and 100 mg/kg BW [43]. These findings indicate that the levels of 5-HMF, 5-MF, and 2-F in coffee products from Chinese markets do not pose health risks. The consumption of 5-HMF-containing food is generally unlikely to be of concern regarding safety. Meanwhile, based on the data collected, it is very challenging to obtain a tolerable daily intake of 5-HMF due to the absence of data on reproductive and developmental toxicity [43]. Most research has paid attention to the health effects of 5-HMF and conducted some experiments in vitro, ex vivo, and in vivo using experimental animals [45,46,47].

There are some factors that may affect the results of the risk assessment of furfural and its derivatives. Only coffee products were selected as the research object, which might have overlooked the estimation of furfural and its derivatives’ exposure. It is important to consider other forms of dietary intake, such as beverages made from dried plums, bread, milk, and foods containing caramel coloring. The ADI of this study was the group ADI of furfural and 11 furfural derivatives [29]. Because our assessment only evaluated a single compound, the results might have biased our estimation of the risk of furfural and its derivatives. Therefore, particular attention should be focused on the co-occurrence and the combined exposure to furfural and all 11 furfural derivatives.

## 4. Conclusions

This is the first study to provide evidence for the occurrence of furfural and its derivatives in coffee products collected in China in 2021. The data presented indicate that the contamination of furfural and its derivatives is common in coffee products in China. However, the levels were lower than in other countries. Coffee beans and powder products have the highest levels of furfural and its derivatives, and 5-HMF was the predominantly accumulated compound, with a 5–10-fold higher level than 5-MF and 2-F. Therefore, it is very important to focus on the exposure to 5-HMF. The main source of exposure for the Chinese consumer is liquid coffee drinks, followed by coffee powder. Among Chinese coffee consumers, the mean dietary exposure of 5-HMF, 5-MF, and 2-F from coffee products was 55. 65, 3.00, and 3.23 μg/kg bw/day, respectively. The risk evaluation results indicate that coffee product intake does not pose a potential health concern among Chinese coffee consumers.

## Figures and Tables

**Table 1:** The selected quantification and fragmented ions used in the analysis.

Analytics	Retention Time (min)	Internal Standard	Quantitative Ion	Qualitative Ion
2-F	8.49	D_4_-2F	96	95.67
D_4_-2-F	8.54	/	100	98
5-MF	9.76	D_4_-2F	110	109.81
5-HMF	14.50	^13^C_6_-5-HMF	97	109.126
^13^C_6_-5-HMF	14.50	/	102	132

**Table 2:** Statistical summary of coffee consumption data among Chinese consumers.

Coffee Category	Consumption of Coffee (g/Day/Person)				
	Mean	P50	P95	P97.5	Max
Coffee beans	7.8	6.7	-	-	15
Coffee powder	4.8	3.3	14.6	17.8	24
Liquid coffee drinks	74.2	60	227.3	278.5	400

P, percentile. Levels less than LOD were considered as 1/2LOD during the calculation of dietary exposure to furan from coffee products.

**Table 3:** Occurrence and contamination levels of furfural and its derivatives in coffee products of China in 2021.

Sample Group	Parameter	5-HMF	5-MF	2-F
Coffee beans (67)	Incidence > LOD (%)	100.0%	100.00%	100.0%
	Mean ± SD(mg/kg)	152.1 ± 207.4	38.5 ± 23.2	49.2 ± 31.8
	Median (mg/kg)	62.3	30.6	43.5
	IQR (interquartile range)	123.9	37.2	36.2
	Maximum (mg/kg)	897.0	96.3	219.0
Coffee powder (42)	Incidence > LOD (%)	100.0%	100.00%	100.0%
	Mean ± SD (mg/kg)	127.7 ± 150.5	38.6 ± 23.7	48.5 ± 29.5
	Median (mg/kg)	83.4	34.2	44.1
	IQR (interquartile range)	92.4	34.2	32.2
	Maximum (mg/kg)	651.0	95.1	122.0
Instant coffee powder (34)	Incidence > LOD (%)	100.0%	97.10%	100.0%
	Mean ± SD (mg/kg)	1479.0 ± 1091.6	16.7 ± 10.8	40.9 ± 31.2
	Median (mg/kg)	1420.0	14.3	35.8
	IQR (interquartile range)	1328.0	15.4	24. 8
	Maximum (mg/kg)	5062.0	41.8	172.0
Drink powder (122)	Incidence > LOD (%)	99.2%	97.50%	100.0%
	Mean ± SD (mg/kg)	326.1 ± 578.2	8.8 ± 21.3	11.8 ± 18.2
	Median (mg/kg)	149.5	2.4	5.6
	IQR (interquartile range)	230.8	2.7	5.1
	Maximum (mg/kg)	3675.0	151	99.0
Package coffee drink (72)	Incidence > LOD (%)	100.0%	76.40%	100.0%
	Mean ± SD (mg/kg)	95.7 ± 710.3	2.8 ± 8.7	2.7 ± 11.4
	Median (mg/kg)	5.5	0.6	0.9
	IQR (interquartile range)	7.0	1.7	1.4
	Maximum (mg/kg)	6035.0	71.4	97.3
Takeaway coffee (112)	Incidence > LOD(%)	95.5%	85.70%	92.9%
	Mean ± SD(mg/kg)	10.7 ± 36.4	2.4 ± 3.6	2.6 ± 5.2
	Median (mg/kg)	2.3	1.4	1.9
	IQR (interquartile range)	4.2	1.9	1.8
	Maximum (mg/kg)	344.0	24.3	52.3

**Table 4:** Estimation of dietary exposure to furfural and its derivatives from coffee products among Chinese consumers.

Group	5-HMF μg/kg bw/Day			5-MF μg/kg bw/Day			2-F μg/kg bw/Day		
	Mean	P95	Range	Mean	P95	Range	Mean	P95	Range
3–6	70.39	-	32.52–108.70	4.17	-	1.93–6.44	4.17	-	1.93–6.44
7–17	48.79	108.91	1.12–119.07	2.79	5.7	0.07–6.45	2.86	6.08	0.07–6.45
≥18	56.33	173.86	1.22–272.67	3.00	9.63	0.07–16.15	3.27	10.39	0.07–16.15
Consumers	55.65	162.96	1.12–272.67	3.00	8.78	0.07–16.15	3.23	9.93	0.07–16.15

Mean: arithmetic mean; P, percentile. Levels less than LOD were considered as 1/2LOD during the calculation of dietary exposure to furan from coffee products.

**Table 5:** The exposure contributions of furfural and its derivatives among Chinese consumers.

Contribution (%)	5-HMF	5-MF	2-F
Coffee beans	0.3	1.3	1.6
Coffee powder	23.7	15.1	20.8
Liquid coffee drinks	76.0	83.6	77.6

## Data Availability

Data are available from the corresponding author upon request.

## References

[1] Albalá-Hurtado S., Veciana-Nogués M.T., Izquierdo-Pulido M., Vidal-Carou M.C. (1998). Determination of free and total furfural compounds in infant milk formulas by high-performance liquid chromatography. J. Agric. Food Chem..

[2] Abraham K., Gürtler R., Berg K., Heinemeyer G., Lampen A., Appel K.E. (2011). Toxicology and risk assessment of 5-hydroxymethylfurfural in food. Mol. Nutr. Food Res..

[3] Kong F., Fan C., Yang Y., Lee B.H., Wei K. (2019). 5-hydroxymethylfurfural-embedded poly (vinyl alcohol)/sodium alginate hybrid hydrogels accelerate wound healing. Int. J. Biol. Macromol..

[4] Shen Z., Ma X., Ali M.M., Liang J., Du Z. (2022). Analysis of the evolution of potential and free furfural compounds in the production chain of infant formula and risk assessment. Food Chem..

[5] Mehrotra S., Rai P., Sharma S.K. (2022). A quick and simple paper-based method for detection of furfural and 5-hydroxymethylfurfural in beverages and fruit juices. Food Chem..

[6] Janzowski C., Glaab V., Samimi E., Schlatter J., Eisenbrand G. (2000). 5-hydroxymethylfurfural: Assessment of mutagenicity, dna-damaging potential and reactivity towards cellular glutathione. Food Chem. Toxicol..

[7] Lee K.G., Shibamoto T. (2002). Toxicology and antioxidant activities of non-enzymatic browning reaction products: Review. Food Rev. Int..

[8] Bauer-Marinovic M., Taugner F., Florian S., Glatt H. (2012). Toxicity studies with 5-hydroxymethylfurfural and its metabolite 5-sulphooxymethylfurfural in wild-type mice and transgenic mice expressing human sulphotransferases 1A1 and 1A2. Arch. Toxicol..

[9] Capuano E., Fogliano V. (2011). Acrylamide and 5-hydroxymethylfurfural (HMF): A review on metabolism, toxicity, occurrence in food and mitigation strategies. LWT-Food Sci. Technol..

[10] Zhang Y.Y., Song Y., Hu X.S., Liao X.J., Ni Y.Y., Li Q.H. (2012). Effects of sugars in batter formula and baking conditions on 5-hydroxymethylfurfural and furfural formation in sponge cake models. Food Res. Int..

[11] Delbecq F., Wang Y., Muralidhara A., El Ouardi K., Marlair G., Len C. (2018). Hydrolysis of hemicellulose and derivatives—A review of recent advances in the production of furfural. Front. Chem..

[12] Hamzalolu A., Gkmen V. (2020). 5-Hydroxymethylfurfural accumulation plays a critical role on acrylamide formation in coffee during roasting as confirmed by multiresponse kinetic modelling. Food Chem..

[13] Ulbricht R.J., Northup S.J., Thomas J.A. (1984). A review of 5-hydroxymethylfurfural (HMF) in parenteral solutions. Fundam. Appl. Toxicol..

[14] Schoental R., Hard G.C., Gibbard S. (1971). Histo-pathology of renal lipomatous tumors in rats treated with the “natural” products, pyrrolizidine alkaloids and a, b-unsaturated aldehydes. J. Natl. Cancer Inst..

[15] Archer M.C., Bruce W.R., Chan C.C., Corpet D.E., Medline A., Roncucci L., Stamp D., Zhang X.M. (1992). Aberrant crypt foci and microadenoma as markers for colon cancer. Environ. Health Perspect..

[16] Bruce W.R., Archer M.C., Corpet D.E., Medline A., Minkin S., Stamp D., Yin Y., Zhang X.M. (1993). Diet, aberrant crypt foci and colorectal cancer. Mutat. Res..

[17] Surh Y.J., Liem A., Miller J.A., Tannenbaum S.R. (1994). 5-Sulfooxymethylfurfural as a possible ultimate mutagenic and carcinogenic metabolite of the maillard reaction product, 5-hydroxymethylfurfural. Carcinogenesis.

[18] Miyakawa Y., Nishi Y., Kato K., Sato H., Takahashi M., Hayashi Y. (1991). Initiating activity of eight pyrolysates of carbohydrates in a two stage mouse skin tumorigenesis model. Carcinogenesis.

[19] Choudhary A., Kumar V., Kumar S., Majid I., Suri S. (2020). 5-hydroxymethylfurfural (hmf) formation, occurrence and potential health concerns: Recent developments. Toxin Rev..

[20] Rufián-Henares J.A., dela Cueva S.P. (2008). Assessment of hydroxymethyl furfural intake in the Spanish diet. Food Addit. Contam. Part A.

[21] Arribas-Lorenzo G., Morales F.J. (2010). Estimation of dietary intake of 5- hydroxymethylfurfural and related substances from coffee to spanish population. Food Chem. Toxicol..

[22] Park S.H., Jo A., Lee K.G. (2021). Effect of various roasting, extraction and drinking conditions on furan and 5-hydroxymethylfurfural levels in coffee. Food Chem..

[23] Gong M., Zhou Z., Liu S., Zhu S., Mao J. (2021). Formation pathways and precursors of furfural during Zhenjiang aromatic vinegar production. Food Chem..

[24] Wang H.Q., Li H.Z., Huang J.L. (2022). Determination of acetaldehyde and furfural in wine by distillation. Food Ind..

[25] Xing Q.Q. (2022). Study on contents of furfural compounds in pasteurized milk. Dairy Ind..

[26] Zhao Z., Li C.Z. (2015). Detection of 5-hydroxymethylfurfural and furfural in dairy products by HPLC. Beverage Ind..

[27] Zhang X.Z., Wu L.H., Gong Z.L., Liu M.M., Shi Y.Y. (2021). Determination of furfural in Baijiu by UV spectrophotometry. Liquor Mak..

[28] Sui H.X., Zhang L., Wu P.G., Song Y., Yong L., Yang D.J., Jiang D.G., Liu Z.P. (2014). Concentration of di(2-ethylhexyl) phthalate (DEHP) in foods and its dietary exposure in China. Int. J. Hyg. Environ. Health.

[29] EFSA (2021). Opinion of the Scientific Panel on Food Additives, Flavourings, Processing Aids and Materials in contact with Food (AFC) on a request from the Commission related to Flavouring Group Evaluation 13: Furfuryl and furan derivatives with and without additional side-chain substituents and heteroatoms from chemical group 14. Scientifific Opinion on Flavouring Group Evaluation 13 Revision 3 (FGE.13Rev3): Furfuryl and furan derivatives with and without additional side-chain substituents and heteroatoms from chemical group 14. EFSA J..

[30] Murkovic M., Bornik M.A. (2010). Formation of 5-hydroxymethyl-2-furfural (hmf) and 5-hydroxymethyl-2-furoic acid during roasting of coffee. Mol. Nutr. Food Res..

[31] Akgün B., Arıcı M. (2019). Evaluation of acrylamide and selected parameters in some turkish coffee brands from the turkish market. Food Addit. Contam. Part A.

[32] Macheiner L., Schmidt A., Karpf F., Mayer H.K. (2020). A novel UHPLC method for determining the degree of coffee roasting by analysis of furans. Food Chem..

[33] Chaichi M., Ghasemzadeh-Mohammadi V., Hashemi M., Mohammadi A. (2015). Furanic compounds and furfural in different coffee products by headspace liquid-phase micro-extraction followed by gas chromatography–mass spectrometry: Survey and effect of brewing procedures. Food Addit. Contam. Part B.

[34] Moon J.K., Shibamoto T. (2009). Role of roasting conditions in the profile of volatile flavor chemicals formed from coffee beans. J. Agric. Food Chem..

[35] Loaeec G., Jacolot P., Helou C., Niquet-Leridon C., Tessier F.J. (2014). Acrylamide, 5-hydroxymethylfurfural and nε-carboxymethyl-lysine in coffee substitutes and instant coffees. Food Addit. Contam. Part A.

[36] Villalón-López N., Serrano-Contreras J.I., Téllez-Medina D.I., Gerardo Zepeda L. (2018). An 1 H NMR-based metabolomic approach to compare the chemical profiling of retail samples of ground roasted and instant coffees. Food Res. Int..

[37] Yu J.M., Chu M., Park H., Park J., Lee K.G. (2021). Analysis of volatile compounds in coffee prepared by various brewing and roasting methods. Foods.

[38] Zhu M., Long Y., Ma Y., Huang Y., Wan Y., Yu Q., Xie J., Chen Y. (2022). Investigation of thermal contaminants in coffee beans induced by roasting: A kinetic modeling approach. Food Chem..

[39] Del Campo G., Berregi I., Caracena R., Zuriarrain J. (2010). Quantitative determination of caffeine, formic acid, trigonelline and 5-(hydroxymethyl) furfural in soluble coffees by 1H NMR spectrometry. Talanta.

[40] Husy T., Haugen M., Murkovic M. (2008). Dietary exposure to 5-hydroxymethylfurfural from Norwegian food and correlations with urine metabolites of shortterm exposure. Food Chem. Toxicol..

[41] Tao D.Y., Hao G., Lu H.X., Tian Y., Feng X.P. (2015). Dental erosion among children aged 3-6 years and its associated indicators. J. Public Health Dent..

[42] Zayed A., Abdelwareth A., Mohamed T.A., Fahmy H.A., Porzel A., Wessjohann Ludger A., Farag Mohamed A. (2022). Dissecting coffee seeds metabolome in context of genotype, roasting degree, and blending in the Middle East using NMR and GC/MS techniques. Food Chem..

[43] Shapla U.M., Solayman M., Alam N., Khalil M.I., Gan S.H. (2018). 5-hydroxymethylfurfural (hmf) levels in honey and other food products: Effects on bees and human health. Chem. Cent. J..

[44] Sachse B., Meinl W., Sommer Y., Glatt H., Seidel A., Monien B.H. (2016). Bioactivation of food genotoxicants 5-hydroxymethylfurfural and furfuryl alcohol by sulfotransferases from human, mouse and rat: A comparative study. Arch. Toxicol..

[45] Jiang Y., Geng N., Wang M., Wu W., Feng N., Zhang X. (2022). 5-HMF affects cardiovascular development in zebrafish larvae via reactive oxygen species and Wnt signaling pathways. Comp. Biochem. Physiol. Part C Toxicol. Pharmacol..

[46] Hou Y.-N., Wang Y.-R., Zheng C.-H., Feng K. (2020). Biotransformation of 5-hydroxymethylfurfural into 2, 5-dihydroxymethylfuran by *Ganoderma sessile* and toxicological assessment of both compounds. AMB Express.

[47] Farag M.R., Alagawany M., Bin-Jumah M. (2020). The Toxicological Aspects of the Heat-Borne Toxicant 5-Hydroxymethylfurfural in Animals: A Review. Molecules.

